# Enrichment of cell cycle pathways in progesterone-treated endometrial organoids of infertile women compared to fertile women

**DOI:** 10.1007/s10815-024-03173-y

**Published:** 2024-07-12

**Authors:** B. N. Bui, A. I. Ardisasmita, F. H. van de Vliert, M. S. Abendroth, M. van Hoesel, S. Mackens, S. A. Fuchs, E. E. S. Nieuwenhuis, F. J. M. Broekmans, G. S. Steba

**Affiliations:** 1 Department of Gynaecology and Reproductive Medicine, University Medical Center Utrecht, Heidelberglaan 100, 3584 CX Utrecht, The Netherlands; 2 Department of Metabolic Diseases, University Medical Center Utrecht, Heidelberglaan 100, 3584 CX Utrecht, The Netherlands; 3 Department of Pediatric Gastroenterology, Wilhelmina Children’s Hospital, University Medical Center Utrecht, Heidelberglaan 100, 3584 CX Utrecht, The Netherlands; 4 Brussels IVF, Universitair Ziekenhuis Brussel, Vrije Universiteit Brussel, Laarbeeklaan 101, 1090 Brussels, Belgium; 5 Department of Science, University College Roosevelt, Lange Noordstraat 1, 4331 CB Middelburg, The Netherlands; 6 Centre for Infertility Care, Dijklander Ziekenhuis, Purmerend, The Netherlands

**Keywords:** Endometrium, Organoids, Transcriptome, RNA-sequencing, Infertility, Fertility

## Abstract

**Purpose:**

To investigate whether the transcriptome profile differs between progesterone-treated infertile and fertile endometrial organoids.

**Methods:**

Endometrial biopsies were obtained from 14 infertile and seven fertile women, after which organoids were generated from isolated epithelial cells. To mimic the secretory phase, organoids were sequentially treated with 17β-estradiol (E2) and progesterone (P4) and subjected to RNA sequencing. Differentially expressed genes (DEGs) were identified using DESeq2 (lfcThreshold = 0, log_2_ Fold Change ≥ 1.0 or ≤ −1.0), and a principal component analysis (PCA) plot was generated. Functional enrichment analysis was performed by overrepresentation analysis and Gene Set Enrichment Analysis (GSEA). To functionally assess proliferation, OrganoSeg surface measurements were performed before (T_0_) and after (T_1_) differentiation of organoids, and T_1_/T_0_ ratios were calculated to determine the proliferation rate.

**Results:**

Although the PCA plot did not show clear clustering of the fertile and infertile samples, 363 significant DEGs (129 upregulated and 234 downregulated) were detected in infertile compared to fertile organoids. Mainly cell cycle processes were highly enriched in infertile organoids. Thus, we hypothesised that proliferative activity during differentiation may be higher in infertile organoids compared to fertile organoids. However, this could not be validated by cell surface measurements.

**Conclusions:**

This study revealed that cell cycle processes were enriched in E2/P4-treated infertile endometrial organoids as compared to fertile organoids. This could reflect persistently higher proliferative activity of the endometrial epithelial cells in differentiated infertile organoids compared to fertile organoids. To confirm this hypothesis, further studies are warranted.

**Supplementary Information:**

The online version contains supplementary material available at 10.1007/s10815-024-03173-y.

## Introduction

Infertility is defined as the failure to achieve a clinical pregnancy after 12 months or more of regular unprotected sexual intercourse [1]. Successful embryo implantation requires a viable blastocyst, a receptive endometrium, and a synchronised interaction between both [2]. In general, blastocysts are considered ‘viable’ when they demonstrate proper development in vitro and thus have the potential for successful pregnancy when transferred into the uterus. However, currently, there is no definitive method available to reliably predict whether an embryo will result in a successful pregnancy.

The endometrium is the inner lining of the uterus and plays a key role in reproduction [3]. The superficial functional layer sheds during menstruation and regenerates in the subsequent cycle, whereas the deeper basal layer harbours the endometrial progenitor stem cells from which regeneration of the endometrium occurs after each menstruation [4]. Histologically, the endometrium is composed of a single layer of luminal epithelial cells, covering a multicellular stromal layer with connective tissue, fibroblast-like stromal cells, tubular glands extending from the luminal epithelium, spiral arteries and immune cells [3]. The cyclic growth, differentiation and breakdown of the endometrium is regulated by the major ovarian sex steroids 17β-estradiol (E2) and progesterone (P4). The menstrual cycle starts with the proliferative phase, which is dominated by follicular E2 production and characterised by extensive proliferation of the endometrium. Following ovulation, P4 is produced by the corpus luteum, heralding the start of the secretory phase, in which the endometrium prepares for implantation [3, 5].

Previous endometrial transcriptome studies based on whole tissue biopsies have identified a vast number of differentially expressed genes (DEGs) in the endometrium of women with recurrent implantation failure (RIF) compared to fertile controls [6–10], suggesting differences in endometrial function. However, there is still an inadequate understanding of the potential role of the endometrium in implantation failure due to the inclusion of heterogeneous study populations, as there is no universally accepted definition of RIF [11]. Furthermore, difficulties to control for exogenous factors in vivo and heterogeneous cellular composition of the endometrial biopsy [12] may have contributed to poor concordance of findings across studies.

Functional endometrial in vivo studies are difficult due to ethical and practical reasons. Therefore, models mimicking the biology of the endometrium are indispensable. Although animal models have provided important insights [13], these do not completely resemble the reproductive tract of humans. Thus, in vitro models of the human endometrium are of great value to further study endometrial function and its role in implantation. The establishment of the three-dimensional (3D) endometrial organoid model has enabled researchers to study endometrial cells that more closely mimic in vivo physiology [14, 15]. Organoids are 3D in vitro structures that self-organise from tissue or stem cells under specific culture conditions and recapitulate the original organ’s key biological properties, such as its function and micro-anatomy [16, 17]. Endometrial organoids have proven to be robustly expandable while recapitulating the original donor’s characteristics and remaining phenotypically and genetically stable over multiple expansions [15], even after tissue cryopreservation [18].

The transcriptome of endometrial organoids has previously been analysed by both bulk and single-cell RNA-sequencing (RNA-seq), which revealed the effects of hormonal treatment on gene expression in endometrial organoids, the existence of several different epithelial cell types as well as important pathways determining the cell fate of these epithelial lineages [19–22]. There are no studies yet comparing the transcriptome of endometrial organoids derived from infertile women versus fertile women, which could provide insight into potential differences in reproductive endometrial function. The objective of this study was to compare the transcriptome profile, analysed by RNA-seq, of endometrial organoids of infertile and fertile women.

## Materials and methods

### Study population

Infertile women were defined as not having reached a clinical pregnancy (i.e. gestational sac visualised by ultrasound) after at least 12 months of regular unprotected intercourse. Endometrial tissue of infertile women was obtained within two randomised controlled trials (RCTs) on endometrial scratching (i.e. biopsy) (SCRaTCH-OFO trial and SCRaTCH trial) [23, 24], as well as an endometrium biobanking study (ENORM study). The full inclusion and exclusion criteria of the two RCTs have been described in detail elsewhere [23, 24]. To summarize, eligible participants for the SCRaTCH-OFO trial [24] were women aged between 18 and 38 years from couples diagnosed with unexplained infertility, who were trying to conceive spontaneously, with a predicted natural conception chance of ≥ 30% by the Hunault model [25]. Whereas for the SCRaTCH trial, eligible participants were women aged between 18 and 44 years who had failed implantation after one full in vitro fertilisation (IVF) or intracytoplasmic sperm injection (ICSI) cycle (i.e. after transfer of fresh and/or frozen embryos) with at least one embryo transfer, and who were planning a new IVF/ICSI cycle [23]. Within the ENORM study, endometrial tissue was obtained from women with RIF, who were defined as having had at least three failed embryo transfers.

Fertile women were defined as having conceived and reached a clinical pregnancy within 12 months of regular unprotected intercourse or intrauterine insemination (IUI) with donor sperm. Endometrial tissue of fertile women was also obtained within the ENORM study. Fertile women were eligible for this study if they fulfilled the following inclusion criteria: (1) aged between 18 and 38 years; (2) no previous infertility or miscarriage and (3) previous spontaneous conception within 12 months with delivery of a healthy baby at term; or undergoing IUI with donor sperm or preimplantation genetic testing (PGT) cycles without ever having conceived before, but not being classified as infertile yet (i.e. not having undergone at least 12 months of attempts) or undergoing IUI with donor sperm or PGT cycles with a previous spontaneous conception within 12 months with delivery of a healthy baby at term. Exclusion criteria were as follows: (1) a history of lower abdominal or pelvic infection; (2) an increased risk of intra-abdominal infection (e.g. due to intestinal surgery in the past or due to the presence of an immunological disease); (3) endometriosis stage III-IV according to the ASRM classification [26]; (4) previous caesarean section with niche formation; (5) the presence of untreated unilateral or bilateral hydrosalpinx; (6) increased risk of bleeding (e.g. the presence of severe coagulation disorders, such as haemophilia); (7) the presence of intrauterine adhesions; (8) previous intrauterine procedures (e.g. curettage, endometrial polyp resection, myomectomy, uterine septum resection); (9) having experienced one or more of the following complications during a previous pregnancy: preterm birth (i.e. before 37 weeks of gestational age), intrauterine growth restriction (i.e. birth weight below the 2.3th percentile), pregnancy induced hypertension, pre-eclampsia, HELLP syndrome, placenta accreta and manual removal of the placenta.

### Endometrial biopsy, tissue processing and cryopreservation

Endometrial tissue was obtained by endometrial biopsy, using an endometrial biopsy catheter (Pipelle catheter). Our protocol for endometrial biopsy has been previously reported [27] and is briefly described below.

Endometrial biopsy was performed either in the mid-luteal phase of a natural cycle, 5 to 8 days after detection of the luteinising hormone (LH) surge by urinary tests (*n* = 16); or in the early luteal phase of a natural cycle (LH + 4; *n* = 1); or during contraceptive pill (*n* = 2) or vaginal ring use (*n* = 1); or during a mock artificial cycle on the 6th day of P4 supplementation (*n* = 1).

After insertion of a speculum, the cervix was cleaned with sterile water. The endometrial biopsy catheter was introduced through the cervix up to the uterine fundus. The piston of the catheter was drawn back to create a vacuum, and the catheter was slowly retracted in a time period of 1 to 2 min while constantly rotating 360°.

After the procedure, the tissue was cryopreserved for organoid development as previously described [18]. Briefly, the tissue was collected in collection medium (Advanced Dulbecco Modified Eagle’s Medium/Nutrient Mixture F-12 (DMEM/F-12) (Thermo Fisher Scientific, USA) with 5% foetal bovine serum (FBS) (Sigma–Aldrich, USA) and 2% penicillin–streptomycin (10,000 U/ml) (Thermo Fisher Scientific) and transported to the laboratory on ice for further processing in a laminar flow hood. After washing with Ca^2+^/Mg^2+^-free phosphate buffered saline (PBS) (Thermo Fisher Scientific) to remove as many blood clots as possible, the tissue was cut into small pieces of 1 mm^3^ and divided into two parts. Both tissue parts were collected in two cryovials containing 1 ml of cryopreservation medium each (Advanced DMEM/F-12 with 30% FBS and 10% dimethyl sulfoxide (DMSO) (Sigma–Aldrich) and slowly frozen to −80 °C overnight in a Mr. Frosty Freezing Container (Thermo Fisher Scientific). Subsequently, the two cryovials were stored in liquid nitrogen at − 196 °C until organoid development.

### Endometrial organoid culture

#### Organoid culture from cryopreserved endometrial biopsies

The cryopreserved endometrial tissue was rapidly thawed at 37 °C and diluted in.

Advanced DMEM/F-12 with 10% FBS (1:10). After centrifugation (at 200 × g for 5 min) and removal of the supernatant, tissue dissociation, organoid culturing and passaging were performed as previously described [15].

In short, endometrial tissue was washed in PBS, further cut into finer pieces and incubated in 1 mg ml^−1^ collagenase IV (Thermo Fisher Scientific) with 10 μM Y-27632 (Merck Millipore, USA) at 37 °C for 1 to 2 h with gentle agitation. Tissue pieces were mechanically triturated every 20 min by pipetting up and down. The digestion was inactivated by 1:1 dilution with Advanced DMEM/F-12 with 10% FBS. The suspension with digested tissue pieces was run over a 40-µm cell strainer (Greiner Bio-One, Austria). The strainer was placed upside down in a petri dish and rinsed with Advanced DMEM/F-12 to remove the endometrial epithelial cells and glandular fragments. The medium was collected from the petri dish, and after centrifugation (200 × g, 5 min, 4 °C), the pellet of endometrial glandular fragments was resuspended in 70% Matrigel (Corning Incorporated, USA) and 30% DMEM/F-12 with 10 μM Y-27632. The cell-Matrigel suspension was plated in a 24-well cell culture plate (Corning Incorporated) with two drops of 15 µl per well. The plate was incubated at 37 °C and 5% CO_2_ for 20 min to allow the Matrigel drops to solidify. Pre-warmed endometrial organoid base culture medium (SupplementaryTable 1) was added with 10 μM Y-27632 once and refreshed every 48 h. After 96 h of culture, the organoid culture medium was supplemented with 1 nM 17β-estradiol (E2) (Sigma–Aldrich) (SupplementaryTable 1) and medium refreshments with E2 organoid culture medium continued every 48 h.

#### Passaging of endometrial organoids

Organoids were passaged every 10–14 days in a ratio of 1:4 to 1:5. Briefly, the organoid-containing Matrigel drops were dissolved in ice-cold Advanced DMEM/F-12, centrifugated (200 × g, 5 min, 4 °C), mechanically triturated, resuspended in 70% Matrigel and 30% Advanced DMEM/F-12, plated in a 24-wells cell culture plate and incubated at 37 °C and 5% CO_2_. Further maintenance of the organoid culture was performed as described above.

#### Hormonal treatment of endometrial organoids

**Fig. 1:**
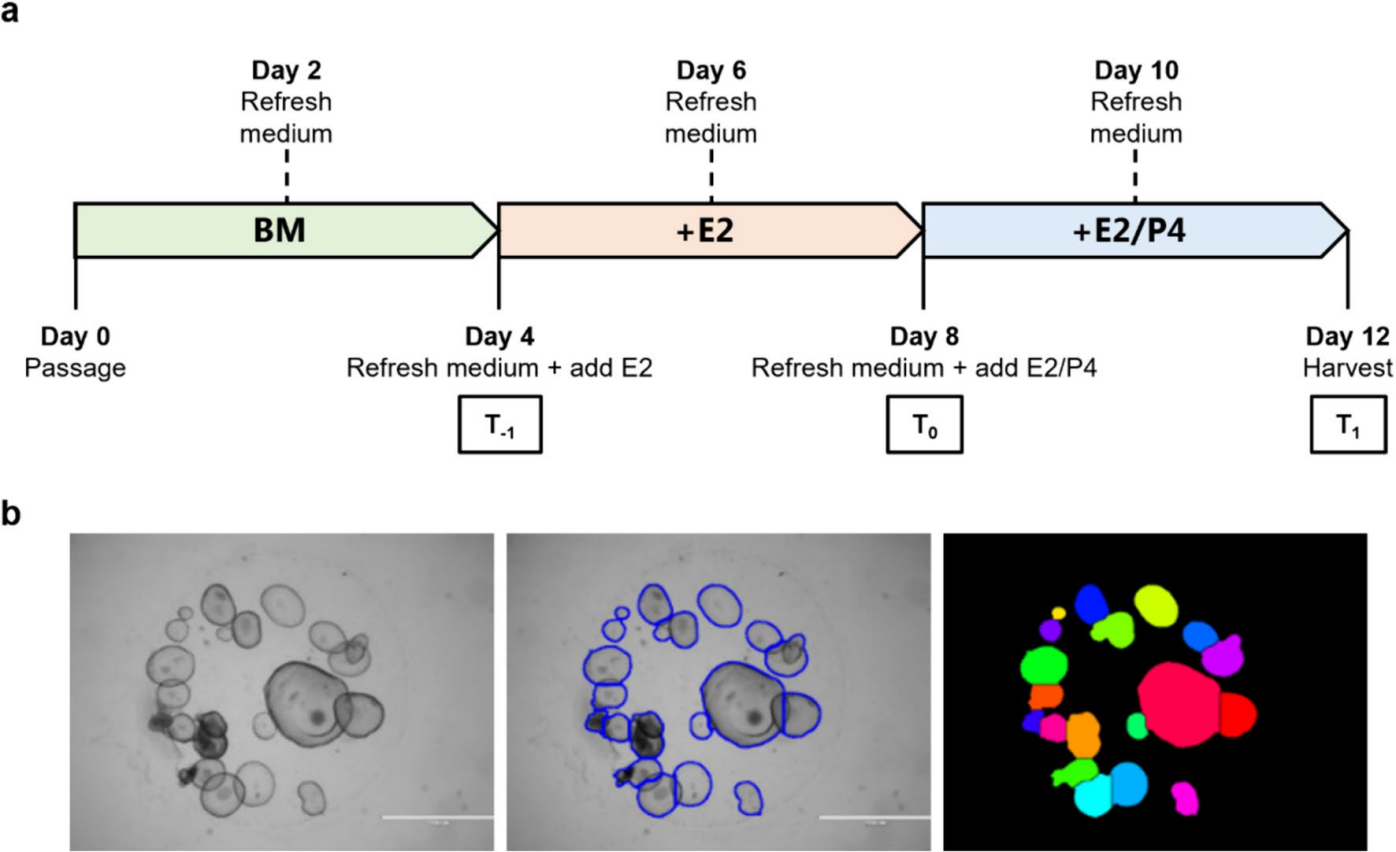
Proliferation assay of infertile and fertile endometrial organoids.** a** Hormonal treatment of endometrial organoids. T_−1_ represented the start of estradiol (E2) supplementation, T_0_ the start of progesterone (P4) supplementation for differentiation of the organoids and T_1_ after differentiation. Photos were taken at the three-time points for evaluation in OrganoSeg. Two organoid lines received a second medium refreshment with E2 medium on days 8–9 and therefore P4 supplementation started 2–3 days later in these lines. BM, base medium; E2, estradiol medium; P4, progesterone medium. **b** Analysis of the two-dimensional surfaces of endometrial organoids in OrganoSeg, using the brightfield images taken at T_0_ and T_1_. The left image shows the original image, the middle image highlights the organoid borders and the right image delineates the measured organoid surfaces

Endometrial organoids between passages 2 and 7 were treated with hormones to mimic the secretory phase for further analysis by RNA-seq (Fig. 1a). During passaging, organoids of each organoid line were seeded in four wells of a 24-well cell culture plate, containing two drops of 15 µl per well. Organoids were first treated with organoid base culture medium without any hormones for 96 h, followed by base medium supplemented with 1 nM E2 for 96 h and finally base medium supplemented with 0.1 nM E2 and 200 ng ml^−1^ P4 (Sigma–Aldrich) for another 96 h (SupplementaryTable 1,Fig. 1a). Organoids were harvested and pooled in two conical tubes of 1.5 ml (Eppendorf, USA). After centrifugation at 200 × g for 5 min, the supernatant was removed and the cell pellets were stored at −80 °C.

### RNA extraction

RNA extraction of the organoid cell pellets was performed by the Utrecht Sequencing Facility (USEQ) (Utrecht, The Netherlands). Cell pellets were thawed and homogenised in RLT Plus lysis buffer (Qiagen). Total RNA extraction was performed using the QIAsymphony SP and the QIAsymphony RNA Kit (Qiagen) following the manufacturer’s protocol. RNA was quantified using the Qubit™ RNA BR Assay Kit (Thermo Fisher Scientific) and the Qubit 4 Fluorometer (Thermo Fisher Scientific). RNA Integrity Number (RIN) was determined using the Agilent RNA 6000 Nano Kit (Agilent Technologies, USA) and the Agilent 2100 Bioanalyzer system (Agilent Technologies).

### RNA-sequencing

RNA-seq was performed by USEQ in three runs. All endometrial organoid RNA samples (*n* = 21 lines in duplicate) had a RIN of ≥ 6.5. Of each sample, an input of 100 ng of total RNA was used to construct a library. Libraries were prepared using the TruSeq Stranded mRNA Kit (Illumina, USA) following the manufacturer’s protocol. xGen Dual Index UMI Adapters (Integrated DNA Technologies (IDT), USA) were used for indexing. Libraries had a final concentration of ≥ 1 ng/µl for sequencing, were pooled equimolarly and single-end (1 × 75 base pairs) sequenced on a NextSeq500 (Illumina).

### RNA-sequencing analysis

Quality control of the raw sequence reads was performed with FastQC (v0.11.8) [28]. TrimGalore (v0.6.5) [29] was used for read trimming based on quality and adapter presence, after which quality control was performed again with FastQC. Ribosomal RNA reads were filtered out using SortMeRNA (v4.3.3) [30]. The resulting reads were aligned to the reference genome hg19 (GRCh37) using the STAR (v2.7.3a) aligner [31]. Follow-up quality control of the mapped (bam) files was performed using Sambamba (v0.7.0) [32], RSeQC (v3.0.1) [33] and PreSeq (v2.0.3) [34]. Read counts were generated using the Subread featureCounts module (v2.0.0) [35]. Batch effect adjustment was performed with ComBat-seq (v3.38.0) [36]. Differential gene expression analysis was performed with DESeq2 (v1.30.1) [37]. Normalised counts were generated by applying DESeq2 variance-stabilizing transformation (VST) to the read counts. To identify significantly differentially expressed genes (DEGs), a lfcThreshold of 0 was used in DESeq2 as a significance threshold, identifying all significantly expressed genes with a log_2_ Fold Change (Log_2_FC) of ≥ 1.0 or ≤ −1.0. Principal component analysis (PCA) was performed using normalised counts and plotted using ggplot2 (v3.3.3) [38].

### Enrichment analysis

Significantly DEGs were subjected to overrepresentation analysis using Enrichr (https://maayanlab.cloud/Enrichr/) [39, 40], annotating Gene Ontology (GO) terms and KEGG (Kyoto Encyclopedia of Genes and Genomes) pathways to gene sets. Enrichr uses the Benjamini–Hochberg method to correct for multiple testing.

In addition, gene set enrichment analysis (GSEA) was performed using the GSEA software from the Broad Institute (v4.1.0, [41, 42]) in order to identify overrepresented GO gene sets. The pre-ranked enrichment method was applied, using a ranked list of DEGs as input for the GSEA-Pre-ranked tool. The DEGs were ranked based on the value obtained from the following formula: sign(log2foldchange) ×  − log10(pval). The analysis was performed using the following parameters: number of permutations = 1000; enrichment statistic = classic; max/min size = 500/15; normalisation method = meandiv. The Normalized Enrichment Score (NES) was used to indicate the degree to which the genes in a gene set were overrepresented.

### Proliferation assay

The proliferation rate was functionally assessed using seven infertile and five fertile lines that had also been selected for RNA-seq. After passaging, the organoid fragments of each organoid line were seeded in 6 wells of a 24-well cell culture plate with a single drop of 20 µl in each well. Organoids were passaged in a ratio of 1:3 to 1:8, depending on the density of the organoids. During seeding of the drops, the wells of the upper and lower row of the cell culture plate were avoided to limit edge effects as much as possible. The organoids were cultured as described above under the “Organoid culture from cryopreserved endometrial biopsies” section.

Photos were taken of all wells using a brightfield microscope (Evos Cell Imaging, Thermo Fisher Scientific) with the 1.25 × objective at the following time points: prior to E2 supplementation (i.e. time point − 1, T_−1_), at the start of E2/P4 supplementation (i.e. T_0_) and at the end of E2/P4 differentiation (i.e. T_1_) (Fig. 1a). Photos were analysed using OrganoSeg, a software programme specifically developed to align spheroid figures in brightfield images of organoids [43] (Fig. 1b). OrganoSeg settings were uniformly configured for all individual wells within an organoid line. However, due to differences in organoid density across the different organoid lines, the settings differed between organoid lines. The surfaces of the organoids in pixels determined at T_0_ and T_1_ were used to calculate a ratio (T_1_/T_0_) to express the proliferation rate during differentiation.

### Statistical analysis

Continuous data were statistically compared between two groups using the independent *t*-test for normally distributed data and the Wilcoxon rank sum test for non-normally distributed data. Categorical data were compared between groups using the chi-square test or Fisher’s exact test in case of an expected frequency of < 5. A *p*-value < 0.05 was considered to indicate a statistically significant difference. Statistical analysis was carried out using SPSS Statistics version 26 (IBM Corporation, USA).

## Results

### Demographics

Endometrial organoids were established from endometrial tissue obtained from seven fertile women and 14 infertile women. Clinical characteristics are summarized in Tables 1 and 2. The median age of both groups was respectively 35 and 34.5 years and did not differ significantly (*p* = 0.84). There were no significant differences in the median BMI and proportion of smokers between both groups (respectively *p* = 0.29 and *p* = 1.00). The infertile group consisted of women with RIF (*n* = 11) and infertile women who had not started ART yet, with thus a relatively good prognosis (*n* = 3) (Table 1). Within the RIF group, primary infertility was the most common type of infertility (81.9%), and the majority was diagnosed with unexplained infertility (45.5%), followed by male factor infertility (36.4%) (Table 1). Among the fertile women, all women had previously conceived within 12 months, and all but one woman had a previous term delivery. The exceptional case involved a woman who had a miscarriage at 10 weeks gestation. One woman (14.3%) had conceived spontaneously and six (85.7%) after donor insemination (Table 2).

**Table 1:** Baseline characteristics of the infertile group

	**Infertile (*n* = 14)**	
Total infertile group	14	(100%)
Median female age (IQR)—yrs	34.5	(31.5–39.0)
Median female BMI (IQR)—kg/m^2^	22.3	(20.7–25.8)
Median duration of infertility (IQR)—mo	31.0	(17.8–57.3)
Smokers	1	(7.1%)
Subgroup: infertile without ART^a^	3	(21.4%)
Median female age (IQR)—yrs	35.0	(32.0–35.5)
Median female BMI (IQR)—kg/m^2^	25.7	(24.6–25.8)
Median duration of infertility (IQR)—mo	18.0	(17.5–24.0)
Smokers	0	
Type of infertility of the female^b^		
Primary	1	(33.3%)
Secondary	2	(66.7%)
Causes of infertility		
Unexplained	3	(100%)
Parity		
Nulliparous	2	(66.7%)
Primiparous	1	(33.3%)
Subgroup: RIF^c^	11	(78.6%)
Median female age (IQR)—yrs	34.0	(32.0–39.0)
Median female BMI (IQR)—kg/m^2^	21.6	(20.6–24.2)
Median duration of infertility (IQR)—mo	36.0	(18.0–58.0)
Smokers	1	(9.1%)
Type of infertility of the female^b^		
Primary	9	(81.9%)
Secondary	2	(18.2%)
Causes of infertility		
Unexplained	5	(45.5%)
Male factor	4	(36.4%)
Ovulatory disorder	1	(9.1%)
Mixed	1	(9.1%)
Parity		
Nulliparous	9	(81.8%)
Primiparous	2	(18.2%)

Data are presented as median (IQR) or number (%)

ART, assisted reproductive technology; BMI, body mass index; mo, months; RIF, recurrent implantation failure; yrs, years.

^a^Defined as women who did not reach a clinical pregnancy after at least 12 months of regular unprotected intercourse and who had not started ART yet.

^b^Primary: female has never conceived before. Secondary: female has conceived before.

^c^Defined as women who have undergone IVF/ICSI with ≥3 failed embryo transfers without clinical pregnancy.

**Table 2:** Baseline characteristics of the fertile group

	**Fertile (*n* = 7)**	
Median female age (IQR)—yrs	35.0	(31.0–38.0)
Median female BMI (IQR)—kg/m^2^	24.2	(22.0–30.1)
Smokers	0	-
Conceived by		
Spontaneously	1	(14.3%)
Donor insemination	6	(85.7%)
Parity		
Nulliparous	1	(14.3%)
Primiparous	5	(71.4%)
Multiparous	1	(14.3%)

Data are presented as median (IQR) or number (%)

*BMI *body mass index, *yrs *years

### Transcriptome profile of infertile vs fertile endometrial organoids

**Fig. 2:**
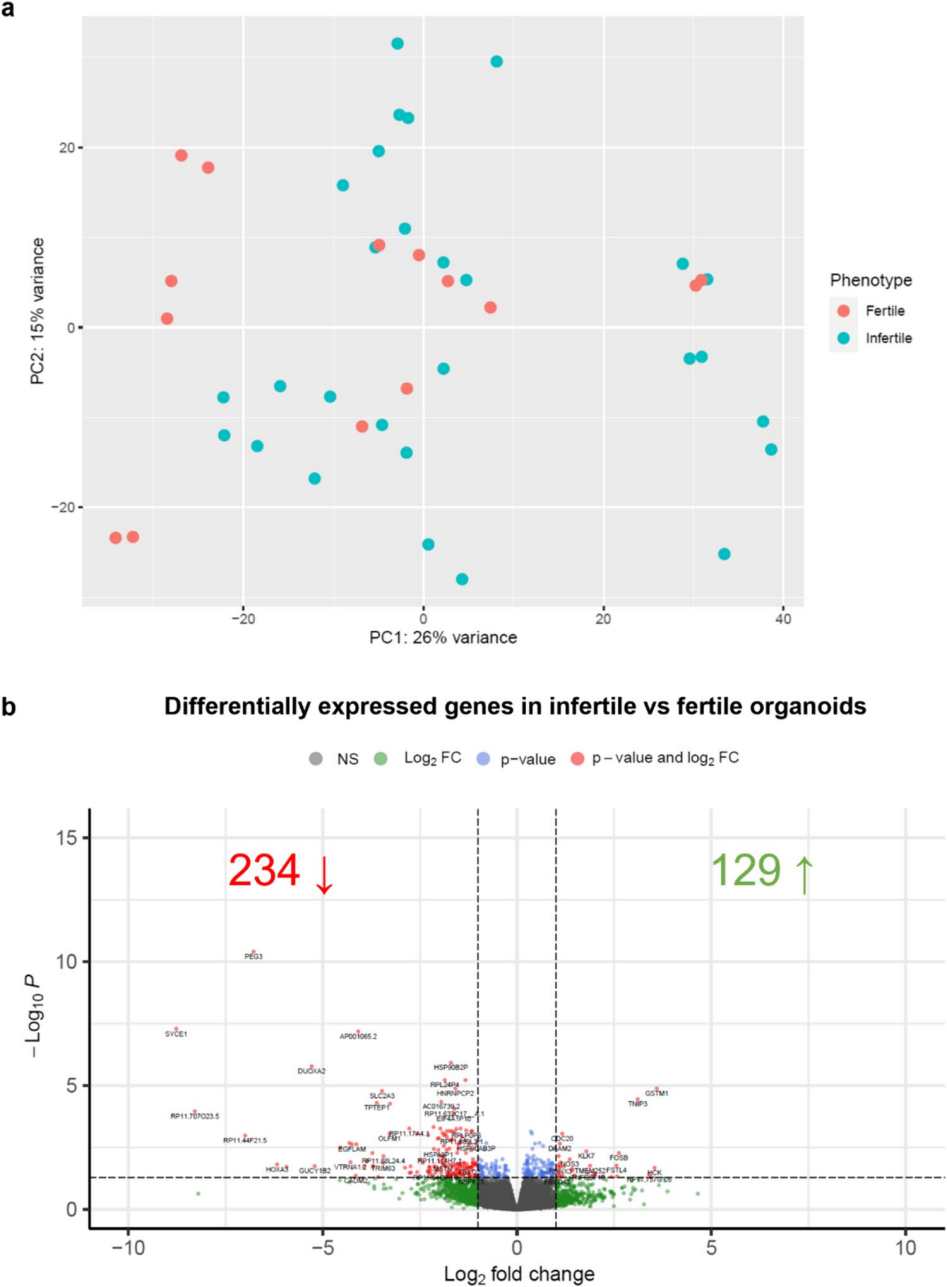
RNA-seq gene expression analysis of infertile vs fertile endometrial organoids. **a** Principal component analysis plot of infertile and fertile endometrial organoid samples after batch effect correction. **b** Volcano plot of differentially expressed genes in infertile organoids compared to fertile organoids. In red are displayed the genes that are significantly differentially expressed with a log_2_ fold change (FC) ≥ 1.0 or ≤  − 1.0 and an adjusted *p*-value (p_adj_) of < 0.05; in green the genes with a log_2_FC ≥ 1.0 or ≤  − 1.0, but with p_adj_ ≥ 0.05; in blue the genes with − 1.0 < log_2_FC < 1.0 and p_adj_ < 0.05; and in grey the genes that are not significantly differentially expressed (i.e. − 1.0 < log_2_FC < 1.0 and p_adj_ ≥ 0.05). NS not significant, FC fold change

Principal component analysis (PCA) did not show clustering of the samples based on clinical phenotype (i.e. fertile or infertile) (Fig. 2a). A total of 363 significant DEGs were identified, of which 129 were upregulated and 234 were downregulated in the infertile samples compared to fertile samples (Fig. 2b,Supplementary Table 2). Additionally, samples did not cluster based on timing of endometrial biopsy, infertility type or infertility diagnosis (Supplementary Figs. 1a-c).

**Fig. 3:**
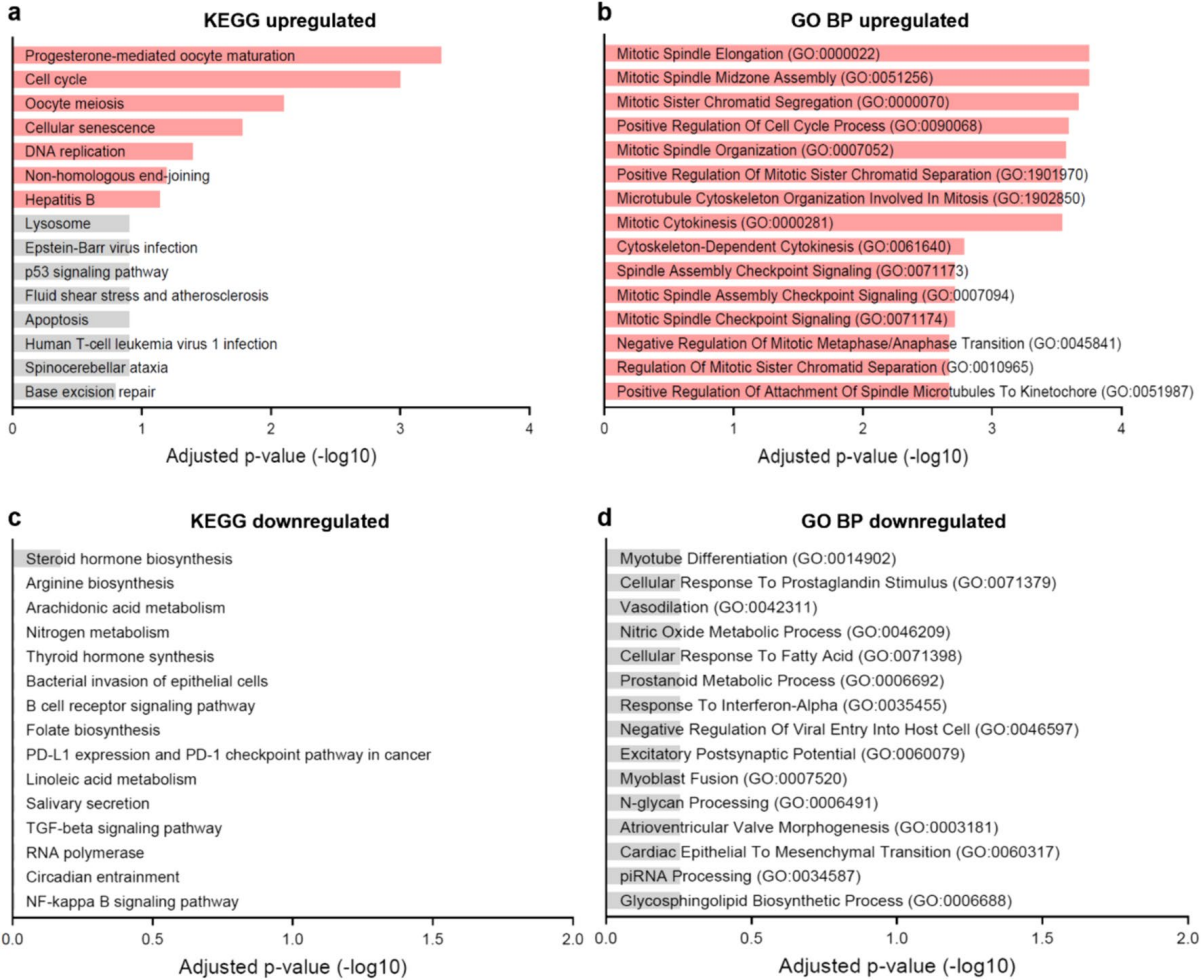
Enrichment analysis annotating significantly differentially expressed genes (DEGs) to top 15 Kyoto Encyclopedia of Genes and Genomes (KEGG) pathways and Gene Ontology (GO) Biological Processes (BP). Pathways and BP are sorted by adjusted *p*-value. Terms with red bars have an adjusted *p*-value of < 0.1 **a** KEGG pathways annotated to significantly upregulated DEGs. **b** GO BP annotated to significantly upregulated DEGs. **c** KEGG pathways annotated to significantly downregulated DEGs. **d** GO BP annotated to significantly downregulated DEGs

Overrepresentation analysis of the significantly upregulated DEGs in infertile endometrial organoids compared to fertile endometrial organoids showed progesterone-mediated oocyte maturation, cell cycle, oocyte meiosis, cellular senescence and DNA replication as the significantly annotated pathways (Fig. 3a,Supplementary Table 3a). Genes that were significantly upregulated in multiple of these pathways were *CCNA2*, *CCNB2*, *CCNB1*, *CDK1*, *AURKA*, *CDC20* and *PCNA*, which all had a function related to the cell cycle. The most significantly GO Biological Processes (BP) annotated to the significantly upregulated DEGs were mitotic spindle elongation, mitotic spindle midzone assembly and mitotic sister chromatid segregation, with *PRC1*, *BIRC5*, *CDCA8* and *KIF23* as overlapping DEGs, which were all linked to cell cycle processes (Fig. 3b,Supplementary Table 3b). Related significant GO Molecular Functions (MF) and Cellular Components (CC) are displayed in Supplementary Tables3c-d, which were also related to cell division. There were no significant pathways or GO BP and GO CC annotated to the significantly downregulated DEGs. As for GO MF, the significant molecular functions annotated to the significantly downregulated DEGs were androsterone dehydrogenase activity and oxidoreductase activity (Supplementary Table 3e).

**Fig. 4:**
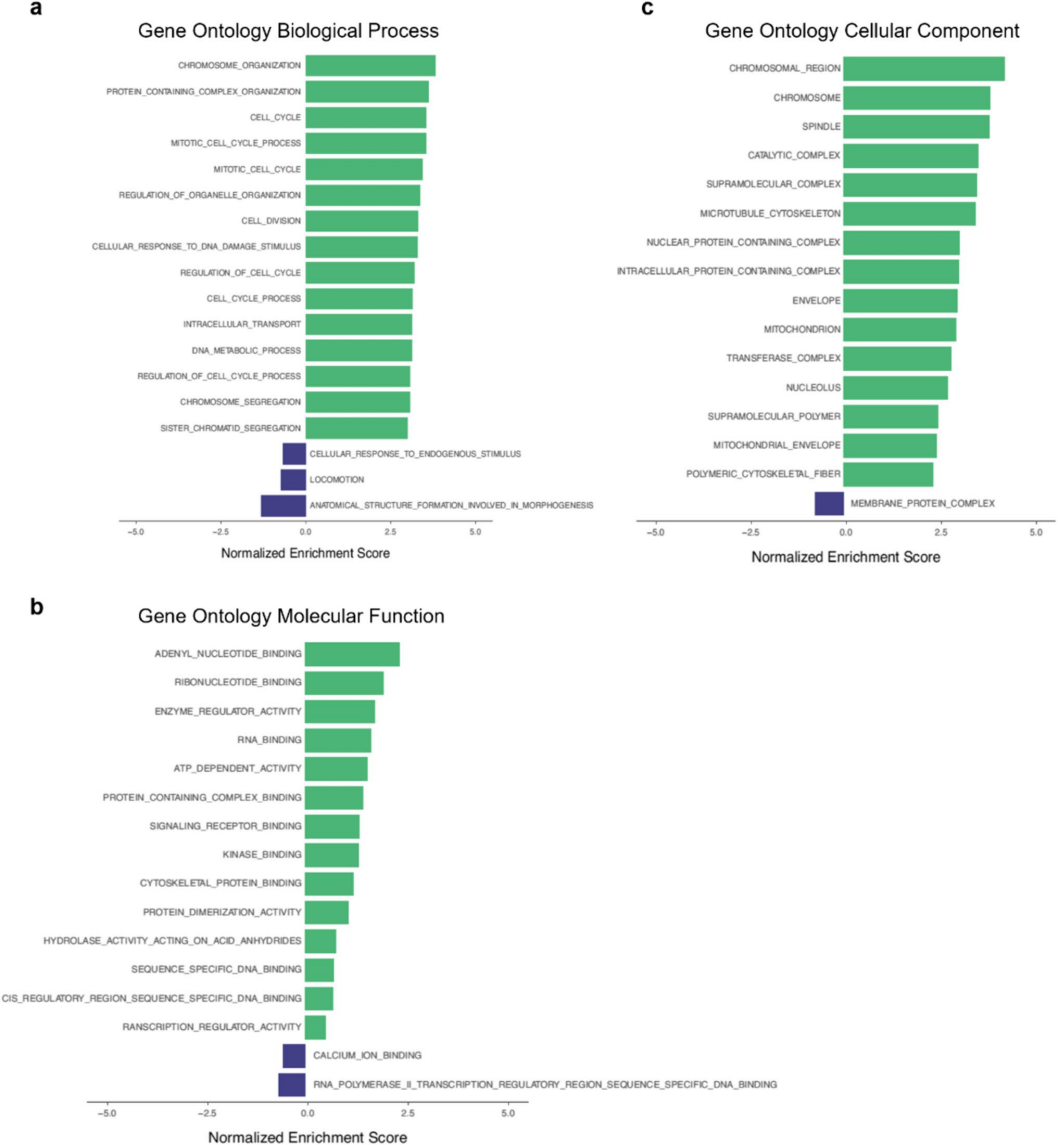
Gene set enrichment analysis (GSEA) of Gene Ontology (GO) gene sets. Biological process (**a**), molecular function (**b**) and cellular component (**c**)

GSEA of GO BP revealed that genes associated with chromosome organisation (NES = 3.81), protein-containing complex organisation (NES = 3.61), cell cycle (NES = 3.55) and mitotic cell cycle process (NES = 3.54) were the most significantly enriched, whereas genes associated with anatomical structure formation involved in morphogenesis (NES =  − 1.32), locomotion (NES =  − 0.73) and cellular response to endogenous stimulus (NES =  − 0.68) were the least enriched in samples of infertile women compared to those of fertile women (Fig. 4a, Supplementary Table 4a), though not significantly. With regards to GO MF, adenyl nucleotide binding (NES = 3.18) and ribonucleotide binding (NES = 2.65) were the most significantly enriched and RNA polymerase II transcription regulatory region sequence-specific DNA binding (NES =  − 0.90) and calcium ion binding (NES =  − 0.75) were the least (not significantly) enriched (Fig. 4b, Supplementary Table 4b). Finally, within the domain GO CC, chromosomal region (NES = 3.51) and chromosome (NES = 3.20) were most significantly enriched, and membrane protein complex (NES =  − 0.63) was not significantly the least enriched (Fig. 4c, Supplementary Table 4c).

### Proliferation assay

**Fig. 5:**
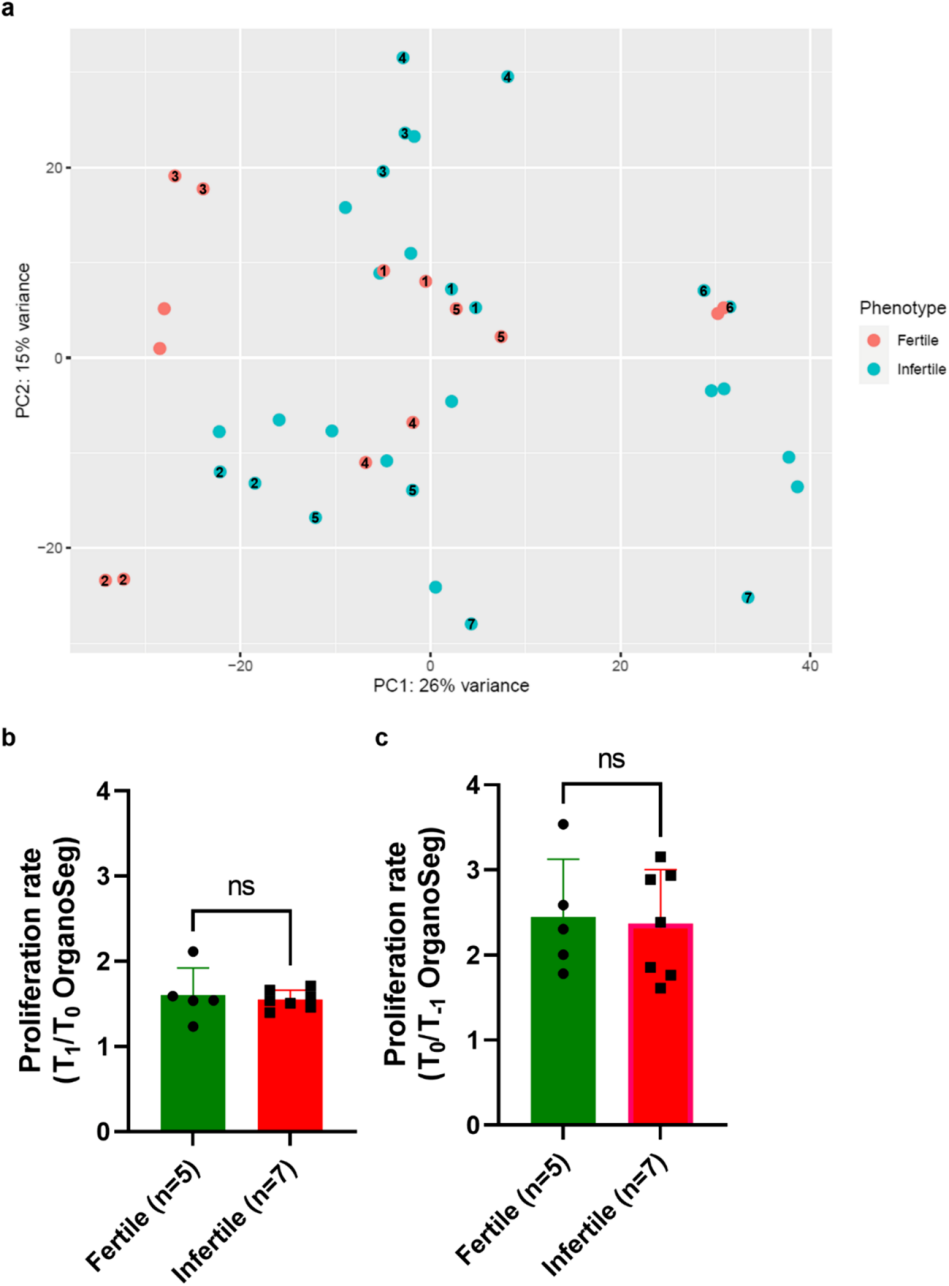
Proliferation assay in infertile vs fertile endometrial organoids. **a** RNA-seq principal component analysis plot with the subset of organoid lines used for the proliferation assay indicated by the numbers (*n* = 7 infertile, *n* = 5 fertile). **b**, **c** Mean proliferation rates in infertile and fertile endometrial organoids expressed as T_1_/T_0_ (**b**) and T_0_/T_−1_ (**c**) OrganoSeg surface measurements

As cell cycle pathways were enriched in differentiated infertile endometrial organoids compared to fertile endometrial organoids, it was hypothesised that infertile differentiated organoids had higher proliferative activity than fertile differentiated organoids. To validate this hypothesis, the proliferation rate of endometrial organoids from both groups under E2/P4 exposure was assessed by OrganoSeg surface measurements at T_1_ and T_0_ (T_1_/T_0_ ratios) (Fig. 5a). The mean T_1_/T_0_ surface ratios were 1.55 and 1.60 in respectively the infertile and fertile group (Fig. 5b). No statistically significant differences in the T_1_/T_0_ ratios between infertile and fertile endometrial organoids (*p* = 0.69) were observed. In addition, no statistically significant differences were demonstrated in proliferation rate between infertile and fertile endometrial organoids prior to differentiation (T_0_/T_−1_ ratios), with a mean surface ratio of 2.37 in infertile organoids and 2.44 in fertile organoids (*p* = 0.85) (Fig. 5c).

## Discussion

### Main findings

In this study, the transcriptome profiles of E2/P4-treated endometrial organoids of infertile and fertile women were compared. Although the PCA did not result in distinct separation of the samples from both groups, a total of 363 genes were significantly differentially expressed between infertile and fertile organoids. Analysis of significant DEGs by overrepresentation analysis as well as by GSEA showed mainly genes related to cell cycle processes to be highly enriched. Assessment of proliferation by surface measurements, right before and after differentiation with E2/P4, did not show any statistically significant differences between infertile and fertile organoids, and could not confirm our hypothesis of a higher proliferative activity during differentiation in infertile organoids compared to fertile organoids.

### Interpretation with regard to previous studies

In accordance with the results of this study, significant upregulation of cell cycle processes in the mid-secretory phase endometrium of infertile RIF patients compared to (fertile) controls was reported in two previous studies in full endometrial biopsies [8, 44]. In concordance with this, increased proliferative activity, determined by Ki67 expression and reduced apoptosis were previously observed in the endometrial glandular epithelial cells of infertile women compared to fertile women [45]. While cell division is an important process during the proliferative phase of the menstrual cycle, proliferative activity is reduced in the in vivo stromal cells of the secretory phase endometrium and completely ceased in the endometrial epithelial cells [46]. In the endometrium of infertile women, the high proliferative activity may be sustained in the endometrial epithelial cells during the secretory phase, leading to inadequate differentiation in preparation to embryo implantation with defective endometrial receptivity, similarly to what has been previously suggested for the eutopic endometrium of women with endometriosis [47–49]. However, rather than upregulation, other studies reported downregulation of the cell cycle pathways in the mid-luteal phase endometrium of infertile RIF patients compared to controls [9, 50], which may suggest that cell cycle activity is altered in various ways in the infertile endometrium. These conflicting findings may also stem from substantial heterogeneity observed across previous endometrial transcriptome studies [51].

No differences in proliferation rate between infertile and fertile endometrial organoids were measured by OrganoSeg. There are several possible explanations for this result. Firstly, it could be possible that the method used was not sensitive enough to detect differences in proliferation. While OrganoSeg surface measurements allow morphometric analysis, this method is not validated yet for the assessment of cell proliferation. Other cell proliferation assays, based on DNA synthesis (e.g. BrdU, EdU assays); metabolic activity of cells (e.g. MTT, XTT assays) or staining of cell proliferation markers (e.g. Ki67) may have yielded different results. Another explanation could be the large heterogeneity within the samples as visualised in the PCA.

This is the first study comparing the transcriptome of infertile to fertile endometrial organoids. Recently, the apical protein secretions of endometrial organoids from infertile and fertile women were compared, showing 150 differentially secreted proteins between both groups, with enrichment in cell membrane movement pathways [52]. In addition, incubation of trophoblast progenitor spheroids with infertile organoid apical secretions significantly impaired the spheroids’ adhesion capacity to organoid epithelial cell monolayers compared to when using the secretions of fertile organoids [52]. These findings show that endometrial organoids are a valuable tool for identifying differences between the infertile and fertile endometrium. However, it is important to bear in mind that interactions with other cell types are also essential to prepare the endometrium for implantation and could modify epithelial cell function [53, 54]. Incorporation of multiple (patient-specific) cell types in the endometrial organoid model may help to mimic the in vivo endometrium but will concomitantly complicate this research system and the interpretation of results. These so-called co-culture models are currently being developed, but still face the challenge of long-term culture [55–58].

### Strengths and limitations

The most important strength of this study is the novelty of comparing the transcriptomes of infertile to fertile endometrial organoids of a relatively large number of women. Endometrial organoids serve as a robust in vitro model, enabling the investigation of endometrial epithelial cells ex vivo under controlled conditions that are consistent across all cell lines. Additionally, gene expression profiling was performed by RNA-seq, a very powerful tool for this purpose due to its high reproducibility and low noise level [59].

Limitations of this study include a heterogeneous group of infertile women, including women with a male factor. This may have contributed to the variability in data within the infertile group and the lack of a clear separation of the infertile and fertile samples in our PCA. There was a similar level of variability within the fertile group, suggesting that the current terminologies to define both fertile and infertile groups may not capture underlying biology. Moreover, variability in endometrial biopsy timing may have contributed to the sample heterogeneity. In addition, endometrial organoids consist of different epithelial cell types [19, 22]. Differences may occur in subpopulations of epithelial cells both within and between organoid lines, which may not be discernible when assessing average gene expression through bulk RNA-seq.

### Clinical implications and/or implications for future research

Previous studies have reported conflicting results concerning the activity of cell cycle pathways in infertile endometrium as compared to fertile endometrium [8, 9, 44, 50]. Endometrial organoids are a representative in vitro model to study the potential role of the endometrium in infertility in a controlled setting. Because epithelial endometrial organoids consist of multiple different epithelial cell types [19, 22], the use of single-cell RNA-seq will shed further light on potential differences in the function of specific endometrial cells between infertile and fertile organoids. A broad omics integrative analysis of both infertile and fertile (co-culture) endometrial organoids may further contribute to identifying biological mechanisms that underlie implantation failure to provide potential targets for future treatments.

## Conclusion

Through transcriptome analysis, enriched cell cycle processes were observed in E2/P4-treated endometrial organoids of infertile women compared to those of fertile women, putatively reflecting higher proliferative activity of differentiated infertile organoids than fertile organoids. Since this could not functionally be confirmed by the OrganoSeg proliferation assay, this hypothesis requires further validation. Future integrative broad omics analysis on the single cell level, including endometrial organoid models, may contribute to improving our understanding of potential differences in the function of infertile and fertile endometrium.

## Supplementary Information

Below is the link to the electronic supplementary material.

Supplementary Material 1.

Supplementary Material 2.

Supplementary Material 3.

## Data Availability

The RNA-sequencing data that support the findings of this study are openly available on Dataverse NL at 10.34894/IHHCKH.
