# Supplementary material for: Enrichment of cell cycle pathways in progesterone-treated endometrial organoids of infertile women compared to fertile women
Source: J Assist Reprod Genet. 2024 Jul 12;41(9):2405–18. doi: 10.1007/s10815-024-03173-y (PMC11405558; doi:10.1007/s10815-024-03173-y)
Supplement: Supplementary file 1 — Supplementary Material 1. [file 10815_2024_3173_MOESM1_ESM.pdf]

Supplementary Figure

Supplementary Figure 1. Principal component analyses of infertile vs fertile endometrial organoids based on several clinical factors. a. Principal component analysis (PCA) plot displaying the timing of endometrial biopsy. HRT, hormone replacement therapy; LH, luteinizing hormone; CD, cycle day; OCP, oral contraceptive pill; VR, vaginal ring. b. PCA plot based on infertility type, displaying primary and secondary infertility samples within the infertile group. NA, not applicable. c. PCA plot displaying the infertility diagnoses among the samples of the infertile group. NA, not applicable.

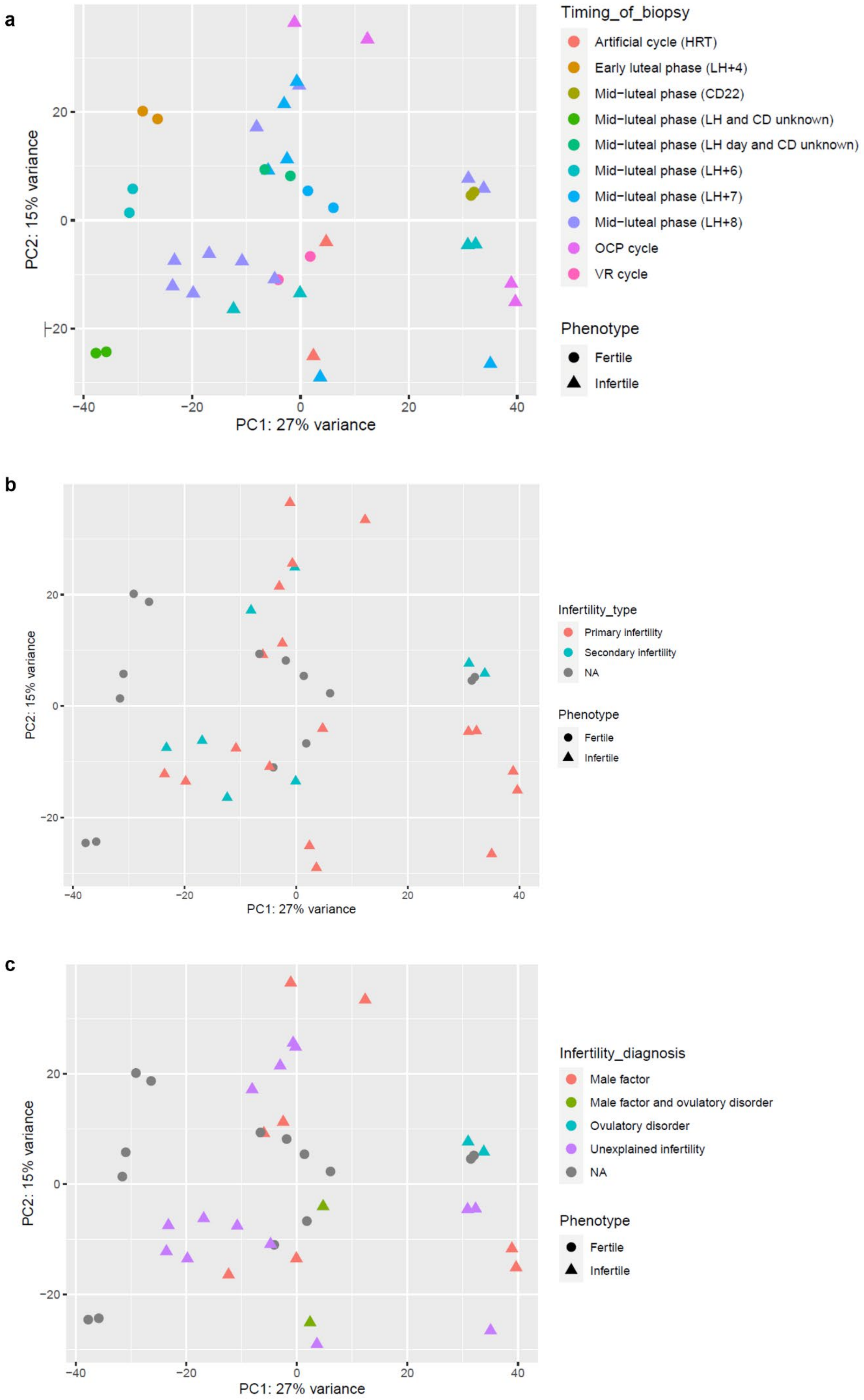
a
Timing_of_biopsy
Artificial cycle (HRT)
Early luteal phase (LH+4)
Mid−luteal phase (CD22)
Mid−luteal phase (LH and CD unknown)
Mid−luteal phase (LH day and CD unknown)
Mid−luteal phase (LH+6)
Mid−luteal phase (LH+7)
Mid−luteal phase (LH+8)
OCP cycle
VR cycle
Phenotype
Fertile
Infertile
PC2: 15% variance
PC1: 27% variance
b
Infertility_type
Primary infertility
Secondary infertility
NA
Phenotype
Fertile
Infertile
PC2: 15% variance
PC1: 27% variance
c
Infertility_diagnosis
Male factor
Male factor and ovulatory disorder
Ovulatory disorder
Unexplained infertility
NA
Phenotype
Fertile
Infertile
PC2: 15% variance
PC1: 27% variance
