# Supplementary material for: Enrichment of cell cycle pathways in progesterone-treated endometrial organoids of infertile women compared to fertile women
Source: J Assist Reprod Genet. 2024 Jul 12;41(9):2405–18. doi: 10.1007/s10815-024-03173-y (PMC11405558; doi:10.1007/s10815-024-03173-y)
Supplement: Supplementary file 2 — Supplementary Material 2. [file 10815_2024_3173_MOESM2_ESM.pdf]

Supplementary Table 1

Supplementary Table 1. Endometrial organoid medium.			
**Product**	**Company**	**Catalogue number**	**Final concentration**
**Base medium**			
Advanced DMEM/F-12	Thermo Fisher Scientific	12634028	1X
Penicillin-Streptomycin	Thermo Fisher Scientific	15140122	Penicillin: 100 units/ml Streptomycin: 100 µg/ml
HEPES	Thermo Fisher Scientific	15630080	10 mM
GlutaMAX	Thermo Fisher Scientific	35050061	1X
R-Spondin-1 conditioned medium	Home made	NA	10%
Noggin conditioned medium	Home made	NA	10%
Recombinant human EGF	Peprotech	100-47	50 ng/ml
Recombinant human FGF-10	Peprotech	100-26	50 ng/ml
Recombinant human FGF basic	R&D Systems	234-FSE-025	2 ng/ml
Nicotinamide	Sigma-Aldrich	N0636	1 mM
N-Acetyl-L-cysteine	Sigma-Aldrich	A9165	1.25 mM
B-27 supplement minus vitamin A	Thermo Fisher Scientific	12587010	1X
N-2 supplement	Thermo Fisher Scientific	17502048	1X
A83-01	Tocris	2939	0.5 µM
SB202190	Sigma-Aldrich	S7067	10 µM
Insulin-Transferrin-Selenium	Thermo Fisher Scientific	41400045	1X
Primocin	Invivogen	ant-pm-2	100 µg/ml
**E2 medium: add to base medium**			
17β-estradiol	Sigma-Aldrich	E2758	1 nM
**P4 medium: add to base medium**			
17β-estradiol	Sigma-Aldrich	E2758	0.1 nM
Progesterone	Sigma-Aldrich	P8783	0.2 µg/ml
E2, estradiol; P4, progesterone; NA, not applicable.			
